# Decentralization and Regionalization of Surgical Care as a Critical Scale-up Strategy in Low- and Middle-Income Countries Comment on "Decentralization and Regionalization of Surgical Care: A Review of Evidence for the Optimal Distribution of Surgical Services in Low- and Middle-Income Countries"

**DOI:** 10.34172/ijhpm.2020.26

**Published:** 2020-02-24

**Authors:** Jaymie A. Henry

**Affiliations:** Department of Surgery, Charles E. Schmidt College of Medicine, Florida Atlantic University, Boca Raton, FL, USA.

**Keywords:** Decentralization, Devolution, Regionalization, Centralization, Essential Surgery, Surgical Scale-Up, Quality, Global Surgery, UHC

## Abstract

As global attention to improve the quality, safety and access to surgical care in low- and middle-income countries (LMICs) increases, the need for evidence-based strategies to reliably scale-up the quality and quantity of surgical services becomes ever more pertinent. Iversen et al discuss the optimal distribution of surgical services, whether through decentralization or regionalization, and propose a strategy that utilizes the dimensions of acuity, complexity and prevalence of surgical conditions to inform national priorities. Proposed expansion of this strategy to encompass levels of scale-up prioritization is discussed in this commentary. The decentralization of emergency obstetric services in LMICs shows promising results and should be further explored. The dearth of evidence of regionalization in LMICs, on the other hand, limits extrapolation of lessons learned. Nevertheless, principles from the successful regionalization of certain services such as trauma care in high-income countries (HICs) can be adapted to LMIC settings and can provide the backbone for innovation in service delivery and safety.

The accompanying article by Iverson et al^1^ details an important narrative-based scoping review on the effectiveness of decentralization and regionalization of surgical services in low- and middle-income countries (LMICs) based on surgical conditions featured in the Disease Control Priorities 3 volume on Essential Surgery using the Donabedian framework of input, process and outcome measures to assess the quality of care provided. The search strategy uncovered 35 studies, the majority of which address the effectiveness of the decentralization of emergency obstetric care and elective procedures such as circumcision and cryotherapy. Studies on the regionalization of surgical services point to the dearth of information available in LMICs as only 20% describe provision of specialized surgical services at a regional hospital addressing a specific condition (eg, cleft lip and palate, obstetric fistula, and cataract extraction), or international visiting specialists providing on-site training at central hospitals or training institutions. The strategies mentioned (eg, short-term missions, international teams providing training or care, professional bodies instituting training) is notable as it is unclear whether they are part of a government-led regionalization strategy or formal public private partnership designed to transfer knowledge and skills to public institutions as opposed to informal engagements from private actors. Nevertheless, the review offers a broad perspective on current efforts to provide surgical services at the district hospital or at a higher-level facility and is a good reference point on the impact of training, international partnerships, and the strengthening of surgical services at various healthcare levels.

The authors propose the dimensions of acuity, complexity, and surgical volume as important considerations in the planning of the distribution of surgical services. This is a useful framework which can be combined in various ways to serve as a practical guide for policy-makers in considering the order of priority for sequential scale up of surgical services whether through decentralization or regionalization. Building off of the authors’ recommendations for decentralizing high acuity, high volume and low complexity procedures while regionalizing low volume, high complexity and low acuity procedures, six other possible combinations are explored and prioritization of the organization of surgical services according to levels are proposed in Table. As cost and volume tend to be key determinants of LMIC prioritization, a slight modification is presented where complexity is considered prior to volume, with acuity as a last factor in ranking the prioritization of services. The rationale is based on the fact that high acuity surgical conditions require more skills and resources to setup and address properly. The following proposal serves as a starting point for further discussion regarding establishing guidelines for the organization of services in LMICs and may be further expanded to include specific interventions.

**Table:** Proposed Matrix for the Prioritization and Organization of Surgical Services in LMICs

**Level**	**Complexity**	**Volume**	**Acuity**	**Example Service**	**Organization**
1	Low	High	High	Basic trauma services Basic obstetric services Basic emergency surgery services (eg, appendectomy)	Decentralized
2	Low	Low	High	Basic emergency surgery services	Decentralized
3	Low	High	Low	Basic general surgery (eg, hernia) Basic ophthalmologic surgery (eg, cataracts)	Decentralized
4	Low	Low	Low	Basic orthopedic service (eg, clubfoot)	Decentralized
5	High	High	High	Complex trauma services	Regionalized
6	High	Low	High	Complex general surgical service (eg, ruptured abdominal aortic aneurysm)	Regionalized
7	High	High	Low	Common cancers (eg, lung cancer)	Regionalized
8	High	Low	Low	Complex oncologic and reconstructive services (eg, pancreatic, liver cancer surgery)	Regionalized

Abbreviation: LMICs, low- and middle-income countries.

Decentralization of health services, especially in developing countries, is complex and multifactorial, and oftentimes depends on the existing political and public administrative structure of the country. ‘Decentralization’ alone invokes certain typologies such as de-concentration, delegation, devolution, and privatization,^2^ which goes beyond mere delivery of surgical services. This distinction is an important consideration in the assessment of the effectiveness of these strategies; however, as the decision to decentralize or regionalize services falls within governments. Thus, the effectiveness of improving certain surgical services goes beyond clinical efficacy since the broader view of health system governance encompasses many other functions such as financing, cost-effectiveness, and management of human resources for health. In practice, certain surgical services may fall within the typologies mentioned. Devolution in Kenya, for example, which involved the transfer of power, roles, and authority from national government to clear and legally recognized geographical boundaries (ie, county governments), was established in 2010 to improve access to health services throughout the country. The Ministry of Health (MoH) remained responsible for leadership and policy development in the health sector, while the counties took on health service financing and provision within their respective jurisdiction. Thus, the establishment of general surgical and obstetric services such as trauma care, C-section capacity, and simple pediatric cases in district and county referral centers falls within the county government while national referral and specialist centers such as cardiac, oncologic, and transplant services falls within the ambit of the MoH.^3,4^ In the Philippines, 25 years of decentralization resulted in multiple tiers of devolved responsibility from the Department of Health to individual local government units. Provinces are responsible for hospitals, municipalities for primary care facilities called rural health units, and cities for both.^5^ Delegation is the transfer of responsibility for decision-making and administration of public functions from the national government to semi-autonomouspublic sector organizations such as hospital corporations.^6^ This may apply to regionalized trauma care provision. Deconcentration redistributes decision-making authority and financial and management responsibilities among different levels of a national government while maintaining existing policies.^6^ Regional referral hospitals handling complex cancer and surgical cases falls within this typology. Privatization involves transferring government responsibility for public services to private institutions such as businesses. Although cogent in developed countries, serious issues were raised regarding this strategy in LMICs outside the cities where the private sector seems to consist of small privately-run clinics and singular faith-based institutions.^7^ Nevertheless, private surgical ambulatory care centers can provide elective general surgery services such as cataract surgery or knee replacement surgery.

To date, strong evidence of the impact and effectiveness of decentralization has yet to be established, especially in LMICs.^8^ No consensus exists on which optimal outcome is assessed given its heterogeneous aspects. Published reviews, however, hint at multiple factors that are required for successful implementation of decentralization such as adequate skills for local counterparts taking on the functions, political will in the capital to implement changes and baseline socio-economic context where decentralization is planned. These factors, along with clarification on what decentralization actually mean in practice according to the different typologies may assist in future assessments of the effectiveness of decentralization of surgical services.^5^ In this review, multiple studies show a decrease in the effect size for the maternal mortality rate and case fatality rates after decentralization and seems to point to powerful evidence of the effectiveness of delivering the right high-quality obstetric services. Further assessment is recommended, however, as effectiveness of quality care delivered includes processes and outcomes, and using the framework from the Lancet Global Health Commission on High Quality Health systems, high-quality care also includes cost-effectiveness.^9^ This can add to the growing body of evidence to strengthen surgical obstetric capacity in lower tiers of maternal care as facility-based births do not necessarily lead to reductions in maternal mortality^10^ while facilities with Cesarean section capacity and high birth volumes (>500/year) were found to have higher quality of care.^11^

Regionalization, on the other hand, has well-demonstrated success in high-income countries (HICs), particularly in trauma care, but does have concerns regarding the cost of transportation and other logistical requirements.^12,13^ The authors do not distinguish between ‘regionalization’ and ‘centralization’ however, but this is an important distinction to make. Other investigators point out that ‘regionalization is not centralization’^12^; rather, a point in the continuum of health governance that seeks to provide ‘intermediary administrative governance and structure to a defined population.’^14^ Thus, the default ‘centralization’ already taking place in less developed surgical systems where most complex surgical services remain in urbanized areas, as the authors rightly point out, may in fact result in potentially lower transportation costs once regionalization effectively takes place. Although specific models from HICs are not directly translatable, certain principles may be adaptable. For example, in the case of trauma system management, essential components include: ‘designation of hospitals with a range of resources, prehospital triage protocols that allow bypassing of non-trauma centers, interfacility transfer agreements, trauma quality improvement programs, ensuring adequate regional coverage, and limited number of designated centers based on need.’^15^ More than ten unique LMIC trauma and emergency medical systems have been reported,^16^ suggesting availability of data that can expand the current review. Regionalization of trauma services in LMICs have generally been the accepted strategy,^16-18^ so perhaps the assessment focus should not be on ‘should we;’ but rather, ‘how’? These same principles can be extrapolated to regionalized maternity centers that focus on high risk pregnancies as being proposed in HICs.^19^

Although clear gains have been established in the regionalization of trauma care, other services are still of unproven benefit. This suggests the need for further expansion of research into the effectiveness of the strategy in LMICs either through inclusion of an extended list of service parameters by which more complex, high-volume interventions make more fiscal sense for the policy-maker to consider setting up such initiatives (eg, cancer, pediatric, neonatal care) in addition to the search for specific procedures listed, an examination of unpublished data or LMIC databases, launching of pilot initiatives designed to demonstrate the effectiveness of regionalization or querying outcomes from public-private-partnerships with existing surgical centers of excellence run by the private sector.^20,21^

The concept of ‘either’ ‘or’ may require an examination of the nuances that exist within a surgical system. Perhaps an exploration of the concept of a ‘continuum’ of care or a ‘hub and spoke model’ where low acuity and low complex surgical services are delivered at point of care (decentralized) while high-risk or high complex cases are identified early and transported or referred to regionalized centers of excellence (regionalization) through an effective referral network system utilizing digital health technology or linked electronic health records is a better representation of the optimal distribution of surgical services.

In short, the current review addresses a critical problem in LMICs that has a potential impact on the scaling up of quality surgical, obstetric, trauma, and anesthesia care through optimal distribution of services given limited resources. The paper highlights important points: (1) Considering surgical acuity, complexity, and volume as parameters in the organization and prioritization of services. This can provide a standardized guide for how MoHs can prioritize the scaling up of such services but should ideally be informed by population-based studies documenting the prevalence of untreated surgical conditions leading to premature mortality or neglected surgical disease, and (2) The importance of workforce training for improved outcomes with adequate quality assurance through partnerships with established institutions both locally and internationally. I would suggest several other points (1) Proven, adaptable principles from decades of HIC experience in the regionalization of trauma care can serve as the backbone for subsequent localization and innovation, (2) Decentralization and regionalization is an inherently political process and needs to be country-led and contextualized according to socioeconomic and geopolitical realities, (3) Establishment of an effective referral network system through a regional ‘hub and spoke’ model with adequate infrastructure to support it can minimize delays in care which can impact outcomes, (4) Partnerships with established non-governmental organizations providing reliable access to surgical care may provide a pathway for knowledge and skills transfer from these groups who have decades of on the ground experience delivering high quality surgical care to governments with nascent experience and needs to be further studied in ways in which it can drive both local and international resources to accelerate the reorganization of services to increase the quality, safety, and access to surgical care, (4) The dearth of LMIC regionalization data is a limiting factor in espousing specific recommendations, and (5) Further studies are needed to guide this critical discussion as LMICs work to increase the volume, quality, complexity, and safety of surgical and anesthesia care for their people.

## Ethical issues

Not applicable.

## Competing interests

Author declares that she has no competing interests.

## Author’s contribution

JAH is the single author of the paper.
